# Coordinated activation of a cluster of MMP genes in response to UVB radiation

**DOI:** 10.1038/s41598-018-20999-6

**Published:** 2018-02-08

**Authors:** Zsuzsanna Ujfaludi, Agota Tuzesi, Hajnalka Majoros, Balint Rothler, Tibor Pankotai, Imre M. Boros

**Affiliations:** 1 Department of Biochemistry and Molecular Biology, FSI, USZ, Közép fasor 52, Szeged, H6726 Hungary; 2 Institute of Biochemistry, BRC, HAS, Temesvári körút 62, H6726 Szeged, Hungary

## Abstract

Ultraviolet (UV) B radiation is a dangerous environmental stressor, which can lead to photoaging, inflammation, immune suppression and tumour formation. A recent report has shown the transcriptional activation of several skin-specific genes including matrix metalloproteases (MMPs) in response to UV irradiation. Here, we use a novel human keratinocyte model, HKerE6SFM, to demonstrate that UVB activates the transcription of most members of the 11q22.3 MMP gene cluster including *MMP13*, *MMP12*, *MMP3*, *MMP1* and *MMP10*. Curiously, the expression of the well-characterized UVB-inducible *MMP9*, which is located outside of the cluster, remains unchanged. In accordance with the increased expression of the MMP gene cluster upon UVB irradiation, RNA polymerase II showed increased occupancy at their promoters following UVB irradiation. The results also demonstrate increased acetylated histone H3K9 levels at the promoters of the *MMP13*, *MMP12*, *MMP3*, *MMP1* and *MMP10* genes. These findings suggest a coordinated transcriptional activation of genes in the MMP cluster at 11q22.3 and that acetylation of histone H3 at lysine 9 has an important role in the UVB-dependent enhancement of transcription of MMP genes in this region.

## Introduction

The epidermis is our first line of defence against direct exposure to environmental stressors such as chemicals or sunlight. Ultraviolet (UV) radiation reaches our skin in the sunlight and has various harmful effects on keratinocytes causing DNA damage, cellular aging, apoptosis or carcinogenesis^1–4^. Solar UV is divided into three categories based on its wavelength, including UVA (320–400 nm), UVB (290–320 nm) and UVC (<290 nm). UVC and the majority of UVB are filtered out by the ozone layer of the atmosphere^5^. Nevertheless, the remaining portion of UVB and UVA cause DNA damage leading to the possible accumulation of mutations initiating skin carcinogenesis^6^. Additionally, UVA and UVB can activate the expression of stress-response and ribosomal genes. UV irradiation also triggers the activation of specific cellular signalling pathways, including those involving the Tumor Necrosis Factor Receptor (TNFR), Epidermal Growth Factor Receptor (EGFR)^7^ and mitogen-activated protein kinases (MAPKs) as a response of reactive oxygen species or direct UV-induced DNA damage^8–10^. The activation of these pathways results in the recruitment of specific transcription factors^11^, which triggers the expression of several downstream genes^12,13^. The induction of these stress-responsive factors triggers various cellular responses such as DNA repair, transcription activation or checkpoint activation^7^. For example, the c-Jun kinases JNK and p38 are activated and the expression of c-Jun is enhanced by UVB through the MAPK pathway leading to c-Jun activation. Subsequently, activated c-Jun forms hetero- or homodimers with other Jun or Fos family members and binds to AP-1 responsive elements in its target genes^14^, such as those encoding matrix metalloproteases^15^. In skin fibroblasts, UVB irradiation enhances both mRNA and protein levels and secretion of MMP1, MMP3 and MMP9^16^. These proteins may activate the cleavage of elastin and collagen compounds of skin connective tissues, which can be repaired under normal circumstances. On the contrary, UVB exposure leads to persistent damage of the extracellular matrix, which finally results in photoaging^17–19^.

In eukaryotic cells, DNA is tightly packed around histone octamers, which enables the genome to fit into the nucleus but provides accessibility for cellular processes regulated by histone posttranslational modifications^20^. Among these modifications is the reversible acetylation–deacetylation cycles of histone N-terminal tails on lysine residues by histone acetyl transferases (HATs) and histone deacetylases (HDACs), respectively^21^. In most cases, histone acetylation results in the relaxation of the chromatin structure to allow access for the transcriptional machinery to DNA, while histone deacetylation compacts chromatin structure^22,23^. The balance between the activities of HATs and HDACs can be shifted by chemicals, such as the HDAC inhibitor trichostatin A, resulting in global hyperacetylation of histones^24^.

UV irradiation causes global changes in the posttranslational modifications of nucleosomal core histones by relaxing the chromatin structure for detection of lesions thereby facilitating DNA repair processes^25–27^. In yeast, acetylation of histone H3 and H4 was increased at the promoter region of a repressed locus upon UVB irradiation^28,29^. In addition to histone acetylation, H2A ubiquitylation as well as H2AX and H3 phosphorylation occur in response to UVB exposure^30–32^. Gene-specific histone acetylation has been associated with UVB-induced transcription in HaCaT cells, indicating that UVB influences the transcriptional response through histone acetylation^33^.

In this study, we have identified and further characterized several UVB-responsive genes using genome-wide and gene-specific approaches and highlight the role of histone H3K9 acetylation in the UVB response of HKerE6SFM cells. The results demonstrate that UVB irradiation is associated with the activation of a specific MMP gene cluster at 11q22.3, which correlates with H3K9ac and RNA Polymerase II (RNAPII) enrichment at MMP promoter regions. These results reveal the coordinated activation of the MMP gene cluster and the strong correlation between UVB-induced transcription upregulation and H3K9 acetylation.

## Materials and Methods

### Cell culture

HKerE6SFM human immortalized keratinocyte cells were kindly provided by Vilmos Tubak (Biological Research Center, Hungarian Academy of Sciences) and were generated as described elsewhere in accordance with the relevant guidelines^34,35^. All experimental protocols were approved by the guidelines of the University of Szeged and the Medical Research Council. The cells were cultured at 37 °C with 5% CO_2_ in Keratinocyte-SFM Medium with L-Glutamine, EGF and BPE (Gibco, Life Technologies) supplemented with 2 mM L-Glutamine (SIGMA) and 1% Antibiotic-Antimycotic Solution (Sigma-Aldrich).

### Cell treatments

UVB irradiation was performed in a sterile chamber containing Vilber Lourmat VL-/6.LM Filtered UV lamps (Vilber Lourmat) positioned at 26 cm above the treatment platform. Wavelengths below 280 nm were eliminated by a cellulose acetate optical filter (kindly provided by Imre Vass, BRC, HAS), which was placed directly under the light source. UVB irradiation dosages were determined by a UVX Digital Ultraviolet Intensity Meter (Cole-Parmer). For UVB treatment, HKerE6SFM cells were grown to ~90% confluence in 60- or 100-mm ∅ dishes. Petri dish covers were removed during the irradiation, and the cells were washed twice with PBS and were finally covered with PBS. Cells were irradiated with 40 or 80 mJ/cm^2^ of UVB, after which the 1XPBS was replaced by supplemented medium, and the cells were incubated for 2, 8 or 24 hours for gene expression measurements and chromatin immunoprecipitation. Histone deacetylase inhibition was performed with chemical treatment using 100 ng/ml trichostatine A (TSA). The effect of TSA on global histone H3 acetylation levels was determined by Western blot analysis (data not shown). For combined treatments, the medium of the cells was replaced between the different steps. Histone acetyltransferase inhibition was performed by adding 30 µM of HATi II inhibitor (Calbiochem #382110) to the cell culture medium immediately after UVB treatment (for 80 mJ/cm^2^). Cells were harvested 24 hours after the irradiation.

### Quantitative real-time PCR (QPCR)

Total RNA samples were prepared with RNeasy Mini Plus Kit (Qiagen) according to the manufacturer’s protocol. For gene expression studies, first-strand cDNA was synthetized using TaqMan^®^ Reverse Transcription Reagents (Applied Biosystems, Life Technologies). Quantitative real-time PCR reactions were performed using the 7500 Real Time PCR System (Applied Biosystems, Life Technologies) and SYBR Green chemistry. Primers (see Table 1) were designed by using Primer3 software^36^. 18S ribosomal RNA was used as the internal control. The Ct value for each studied mRNA was normalized to the internal control, and the alterations in expression levels of the examined genes were calculated by the ΔΔCt method^37^. The mean and standard deviation were calculated from three independent sample sets. P-values were calculated using paired ANOVA. The calculation of the qPCR results from the immunoprecipitated DNA samples is described in the *Chromatin Immunoprecipitation* section.

**Table 1:** Sequences of primers used for qPCR reactions.

Gene	Forward primer (5′-3′)	Reverse primer (5′-3′)
h18SRNS	AAACGGCTACCACATCCAAG	CGCTCCCAAGATCCAACTAC
ATF3	CTCCTGGGTCACTGGTGTTT	GCTACCTCGGCTTTTGTGAT
COX2	TGAGTGTGGGATTTGACCAG	TGTGTTTGGAGTGGGTTTCA
MMP13	GATGCAGGCGCCAGAAGAATCT	CAAAAACGCCAGACAAATGTGACC
MMP12	GATGCACGCACCTCGATGT	GGCCCCCCTGGCATT
MMP3	GGCAGTTTGCTCAGCCTATC	TCACCTCCAATCCAAGGAAC
MMP1	GATGTGGAGTGCCTGATGTG	CTGCTTGACCCTCAGAGACC
MMP10	CATACCCTGGGTTTTCCTCCAA	GTCCGCTGCAAAGAAGTATGTTTTC
MMP9	GGGAAGATGCTGCTGTTCA	TCAACTCACTCCGGGAACTC
MMP13 inic	TGACTGGGAAGTGGAAACCT	CGACAATGAGTCCAGCTCAA
MMP12 inic	GCTTTTGTTTGCATGTTTTTGA	AAGAGCTCCAGAAGCAGTGG
MMP3 inic	CCATTTGGATGAAAGCAAGG	GGATAGGCTGAGCAAACTGC
MMP1 inic	GCAACACCAAGTGATTCCAA	AGCTGTGCATACTGGCCTTT
MMP10 inic	GAAAGGCCACTAGGGGGTAG	TTGGAGTCCTCCTCTTTTGC
MMP9 inic	GAGTCAGCAACTTGCCTGTCA	GCTTCCAGGGAAGAGCACAA

### Microarray

Total RNA samples from control, UVB-, TSA- and UVB + TSA-treated HKerE6SFM cells were prepared as described above. Each category represented three independent experimental triplicates. RNA integrity was checked on an Agilent Bioanalyzer 2100 (Agilent Technologies), and RNA samples with RIN values >9.0 were used in further experiments. A UV spectrophotometer, NanoDrop ND-1000 (Thermo Scientific), was used to determine RNA concentrations.

The global expression pattern was analysed on GeneChip^®^ Human Gene 1.0 ST arrays (Affymetrix). The Ambion WT Expression Kit (Life Technologies) and GeneChip WT Terminal Labeling and Control Kit (Affymetrix) were used for amplifying and labelling 250ng of total RNA samples according to the manufacturer’s protocol. Samples were hybridized at 45 °C for 16 hours, and subsequently washed by a standard protocol using the Affymetrix GeneChip Fluidics Station 450. The arrays were scanned on the GeneChip^®^ Scanner 7 G (Affymetrix). RNA labelling and hybridization was processed by UD-GenoMed Medical Genomic Technologies Ltd. (Debrecen, Hungary). Analyses were performed by using GeneSpring^®^ GX7.3.1 (Agilent) software. Raw data (CEL files) were analysed by using the RMA algorithm. Data were normalized using per-chip normalization (global scaling). Genes (probe sets) with a low expression (raw expression <100) were filtered first. Next, gene sets with normalized expression <−0.5 or >0.5 were sorted in all samples. Gene groups were determined using one-way ANOVA, which showed a significant difference with P values < 0.05 at least in one of the four conditions (Multiple Testing Correction: Benjamini and Hochberg False Discovery Rate). Genes showing significant expression alterations for the different treatments were selected by comparing the categories to each other (control vs. UVB, control vs. TSA, control vs. UVB + TSA and UVB vs. UVB + TSA). A heat map including these genes was generated by Heatmapper online software using an average linkage algorithm with the Elucleidean distance method for clustering. The microarray data have been deposited in the ArrayExpress database at EMBL-EBI (www.ebi.ac.uk/arrayexpress) and are available under accession number E-MTAB-6288.

### Chromatin immunoprecipitation

HKerE6SFM cells were grown to 90% confluence and cross-linked in 1% formaldehyde at room temperature for 8 min. The cross-linking was stopped by the addition of 1/20 volume of glycine and incubation at room temperature for 5 min. The cells were washed three times with ice-cold PBS, collected and then pelleted by centrifugation at 2950 × g for 5 min at 4 °C. The cell pellets were lysed in ice-cold swelling buffer (25 mM HEPES, 1.5 mM NaCl, 10 mM KCl, 0.1% Nonidet P-40, 1 mM DTT, 1 mM PMSF, 10 μg Aprotinin, 10 μg Leupeptin) for 10 min on ice. Cell nuclei were pelleted at 2950 × g for 5 min at 4 °C and resuspended in ice-cold sonication buffer (50 mM HEPES, 140 mM NaCl, 1 mM EDTA, 1% Triton X-100, 0.1% Na-deoxycholate, 0.1% SDS, 1 mM PMSF, 10 μg/ml Aprotinin, 10 μg/ml Leupeptin) and were incubated on ice for 45 min. Chromatin samples were sonicated with five pulses of 30 s ON and 1 min OFF cycles at high power settings (Diagenode). Chromatin concentrations were determined by NanoDrop ND-1000 (Thermo Scientific), and 150 μg chromatin of each category was pre-cleared with blocked protein A sepharose beads (Sigma-Aldrich) at 4 °C for 2.5 hours and aliquoted. Antibodies recognizing H3 (Ab1791, Abcam) or H3K9ac (Ab4441, Abcam) or RNAPII (PolII7G5, kindly provided by Laszlo Tora, IGBMC, Strasbourg) were added to the chromatin aliquots, and rotated at 4 °C for 16 hours. Two of the aliquots of each category served as total input controls (TIC) and no antibody controls (NAC), respectively. Antibody-chromatin complexes were bound to 20 μl of blocked Protein A sepharose beads for 4 hours at 4 °C. Beads were collected at 470 × g for 5 min at 4 °C. The supernatants of the TIC/NAC samples served as total input controls. Beads were washed at 4 °C five times with sonication buffer, then with LiCl containing buffer (250 mM LiCl, 0.5% Nonidet P-40, 0.5% Na-deoxycholate, 1 mM EDTA, 10 mM Tris pH8.0) and then with TE buffer (10 mM Tris HCl pH 7.5, 1 mM EDTA pH 8.0). Finally, the beads were resuspended in TE. RNA contamination was removed by the addition of 0.1 mg/ml RNaseA to each sample and incubation at 37 °C for 30 min and then at 65 °C for 16 hours. The immunoprecipitated samples and TICs were reverse cross-linked by the addition of 0.5 mg/ml of Proteinase K (Merck, Sigma-Aldrich) and 0.5% SDS and incubated for 3 hours at 50 °C. DNA was extracted with phenol-chloroform-isoamyl alcohol and precipitated by ethanol. DNA pellets were resuspended in TE and stored at −20 °C. The amounts of immunoprecipitated DNA were calculated as the % of total input controls, normalized to H3 control samples. Quantitative real-time PCR reactions were performed with primers specific for the transcription initiation sites of MMP genes as indicated in Table 1.

### Statistics

Statistical analysis was performed on paired gene groups from different experimental setup by using one-way ANOVA to analyse the differences among group means. Significant difference with P values < 0.05 represents a single star, while two stars represent P < 0.01, triple star P < 0.001 while four stars P < 0.0001 (Multiple Testing Correction: Benjamini and Hochberg False Discovery Rate).

## Results

### Histone deacetylase inhibitor, TSA influences the UVB-dependent transcriptional activation of *ATF3* and *COX2 in* HKerE6SFM cells

UVB irradiation causes alterations in the gene expression profile of epidermal cells in a dose-dependent manner^38^. HaCaT cells, the frequently used model of keratinocytes, are aneuploid and carry two point mutations that result in amino acid substitutions in the DNA-binding domain of p53^39^. Additionally, the cellular level of p53 is high in HaCaT cells due to the extended half-life of the protein. Therefore, HKerE6SFM cells were used instead to study UV-induced effects. This human keratinocyte cell line was established by immortalization of primary keratinocytes by HPV and maybe a more suitable model to study DNA damage-triggered cellular responses. In order to examine the kinetics of UVB-induced transcription activation in the HKerE6SFM human keratinocytes, the expression levels of two known UVB-induced genes were measured including, *activating transcription factor 3 (ATF3)* and *cyclooxygenase 2 (COX2)*^12,38,40,41^. HKerE6SFM cells were irradiated with 40- or 80-mJ/cm^2^ doses of UVB followed by the monitoring of *ATF3* and *COX2 *mRNA levels at 2, 8 and 24 hours. The transcription of both genes was significantly upregulated upon irradiation with a high dose of UVB; however, the kinetics of the gene expression induction differed for the two genes. The mRNA level of *ATF3* first showed a rapid accumulation, and then it reached a steady-state level (Fig. S1). In contrast, the rapid increase of *COX2* mRNA levels suggested a more robust transcriptional activation, which then decreased and remained nonetheless induced 24 hours after irradiation as compared to the control (Fig. S1). Treatment with the lower dose of UVB (40 mJ/cm^2^) resulted in weaker induction of *COX2*, while it did not affect *ATF3* mRNA levels. Thus, the results of qPCR experiments showed that *ATF3* and *COX2* mRNA levels were elevated in a dose-dependent manner in response to UVB in HKerE6SFM keratinocytes.

**Figure 1:**
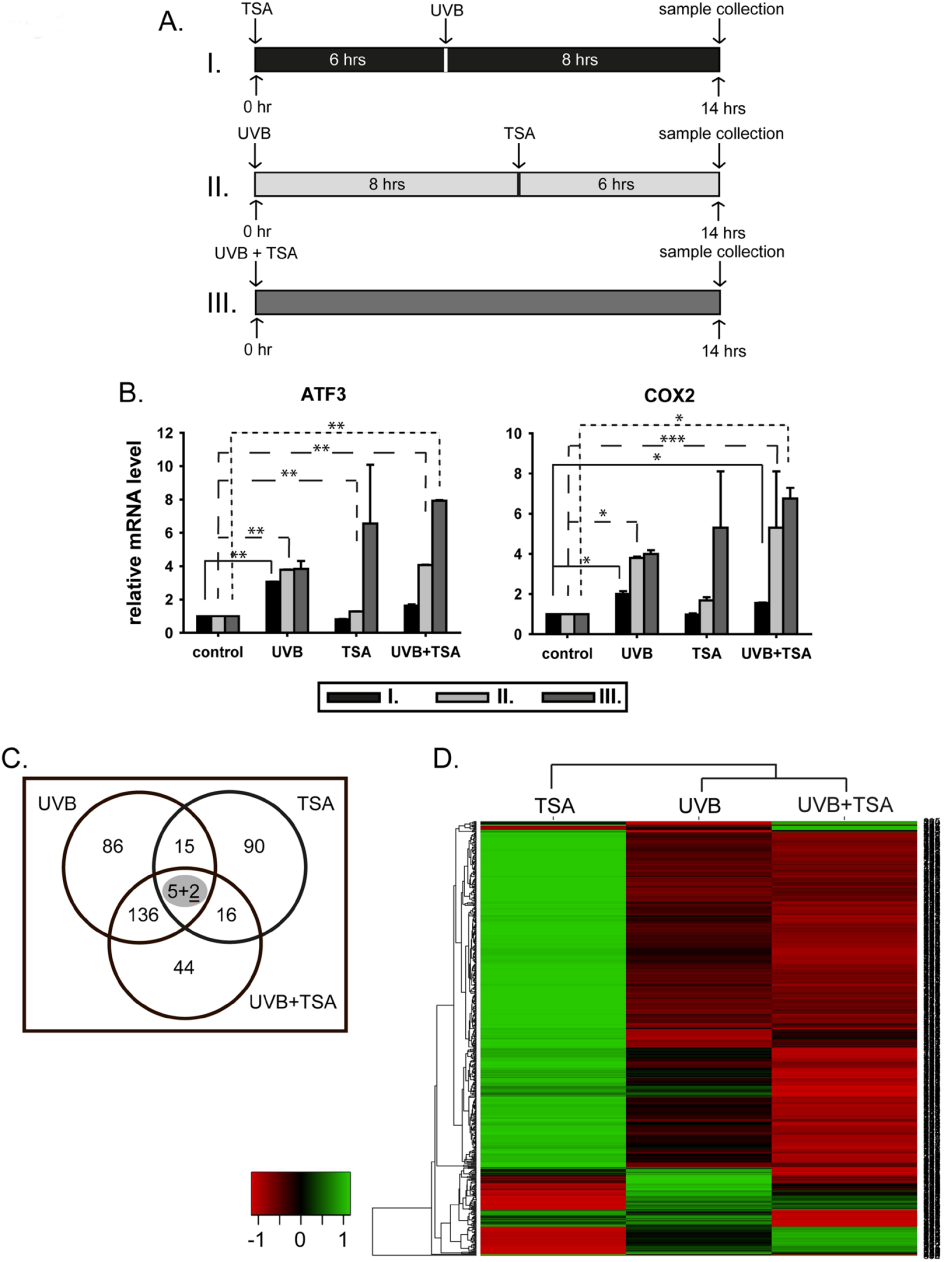
UVB-induced expression level of *ATF3* and *COX2* influenced by TSA. HKerE6SFM cells were treated with high doses of UVB and HDAC inhibitor TSA in different combinations. (**A**) Treatment combinations are illustrated in panel A, rows I, II and III. (**B**) The mRNAs were isolated 14 hours after the first treatment and the expression levels of *ATF3* and *COX2* were analysed by qPCR. The means and standard deviations of five independent experimental triplicates are indicated as fold-expression compared to the control. Standard deviations and statistical significances are shown. Star indicates statistical significance between the dataset (paired Anova *P < 0.05, **P < 0.01, ***P < 0.001, ****P < 0.0001). Legends for the X-axis are *control* (no treatment), *UVB* (only UVB no TSA), *TSA* (only TSA no UVB) and *UVB* + *TSA* (both UVB and TSA). Graph legends (I, II and III.) correspond to UVB and TSA treatment indicated in Panel A. (**C**) Plot of genes differentially expressed in HKerE6SFM cells upon UVB, TSA or UVB + TSA combined treatment in microarray experiments. Circles represent groups of genes modified by UVB, TSA and UVB + TSA treatments compared to the control, respectively. Numbers indicate the number of genes showing altered mRNA levels by one or more stimuli. Underlined, bold number: superinduced genes in UVB + TSA samples (compared to UVB treated samples). (**D**) Heatmap representation of differential gene expression following UVB and TSA treatment. Only genes displaying alterations upon one or the other treatment are included in the map. Red and green colour indicates down- and upregulation, respectively. Expression changes are shown in a log_2_ scale.

Next, the effects of histone acetylation levels on UVB-induced transcriptional activation were assessed in HKerE6SFM cells. Recently, Pollack *et al*. demonstrated that UVB-induced histone acetylation in the HaCaT keratinocyte cell line is associated with UVB-dependent transcriptional responses. In addition, the results showed that a histone deacetylase inhibitor triggered the superinduction of several genes mimicking the effect of multiple UVB exposures^33^. To investigate the role of histone acetylation in response to UVB exposure in HKerE6SFM cells, we established different multi-step treatment protocols (Fig. 1A. upper panel). A high dose of UVB (80 mJ/cm^2^) was administered along with histone deacetylase inhibitor and TSA in different combinations, followed by the determination of *ATF3* and *COX2* mRNA levels (Fig. 1A. lower panel). The expression of both *ATF3* and *COX2* mRNA was induced by UVB and TSA. Exposure to UVB resulted in a rapid 4–5 fold induction in the level of *ATF3* and *COX2* mRNA, which was fairly independent of whether the radiation treatment preceded the mRNA detection by 8 or 14 h (Fig. 1, part A, UVB columns). In contrast, significant accumulation of *ATF3* and *COX2* mRNA (7- to 10-fold increase) was observed only when treatment with the HDAC inhibitor TSA was applied shortly before mRNA detection (Fig. 1, part A, TSA columns). In combination, when TSA was applied prior to UVB exposure (Fig. 1A, row I.), *ATF3* and *COX2* mRNA levels were lower than those in UVB-treated cells (Fig. 1A, lower panel, black bars). The addition of TSA at eight hours after UVB irradiation was associated with higher *ATF3* and *COX2* mRNA levels (Fig. 1A, row II.) than those in UVB-treated cells (Fig. 1A, lower panel, light grey bars). Therefore, the simultaneous exposure of HKerE6SFM cells to UVB and TSA resulted in transcriptional superactivation of the *ATF3* and *COX2* genes (Fig. 1A, UVB vs. UVB + TSA), thus, corroborating the data published by Pollack *et al*.^33^. These findings suggest that histone acetylation plays a role in the UVB-mediated transcriptional response of HKerE6SFM cells. Since TSA inhibits HDAC activity, UVB-induced histone acetylation might be shifted towards a hyper-acetylated state that results in the superinduction of *ATF3* and *COX2*.

### TSA modifies the UVB-induced transcriptional activation of several genes in HKerE6SFM cells

To identify genes in which UVB-induced transcription activation may involve the alteration of histone acetylation, the global expression profile of untreated HKerE6SFM cells (control) was compared to that of cells exposed to UVB, TSA or UVB + TSA using Affymetrix microarray technology (Fig. 1A). HKerE6SFM cells were irradiated with UVB prior to TSA exposure to trigger the robust transcriptional activation of *ATF3* and *COX2* mRNA (Fig. 1B and Supplementary Fig. 1). The number of differentially expressed genes after UVB, TSA or UVB + TSA treatments were 244, 128 and 203, respectively (Fig. 1C and D). UVB, TSA and their combination activated the transcription of a partially overlapping set of genes, among which only seven showed similar changes of activation under all three conditions examined (Fig. 1C). Interestingly, two of these genes (*MMP13* and *MMP12*) were members of the matrix metalloprotease gene family.

**Figure 2:**
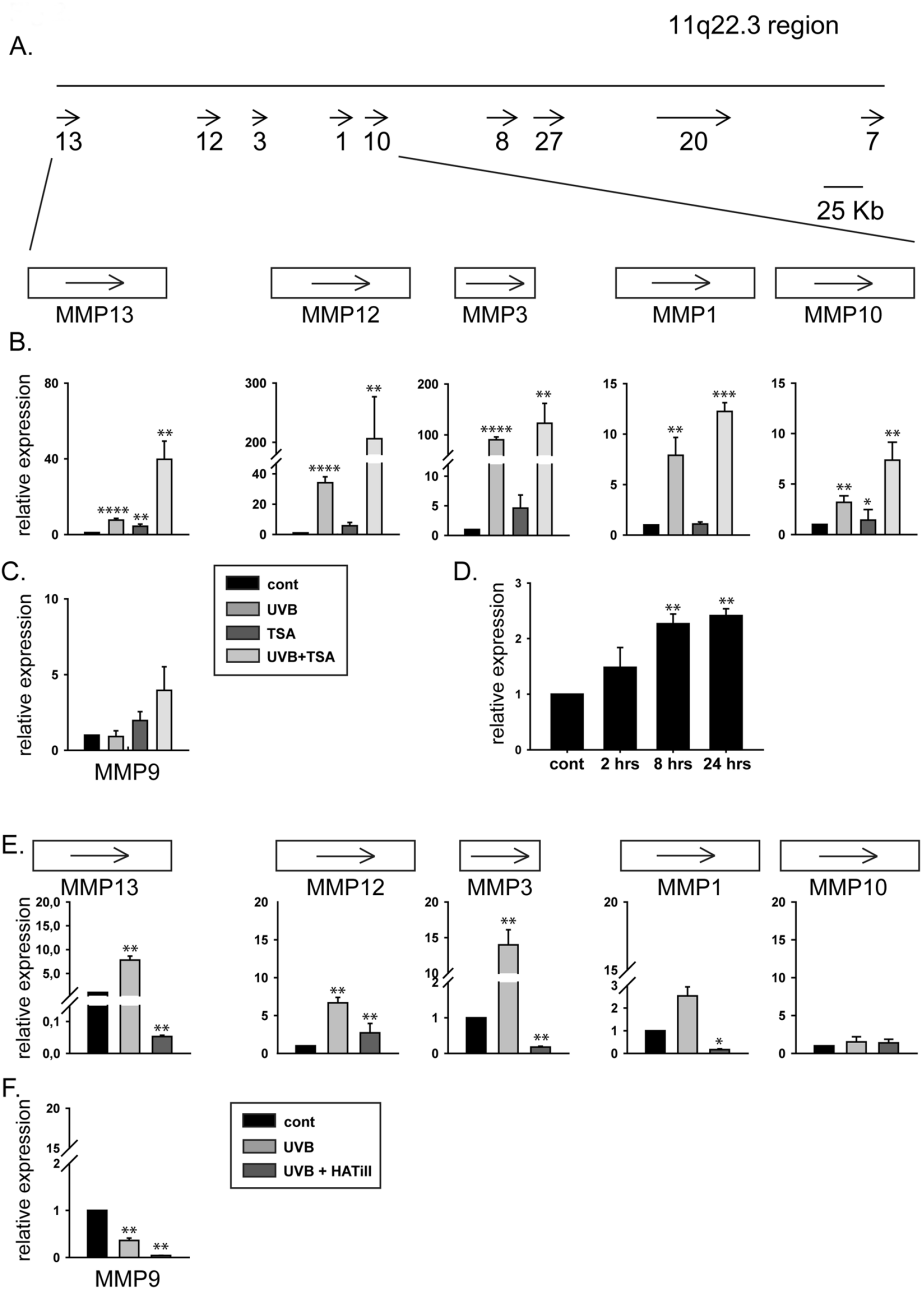
Differential expression of MMP genes located in the 11q22.3 region and *MMP9*. (**A**) Schematic drawing of the MMP gene cluster localized at chromosome 11q22.3 based on the NCBI GeneBank database. Arrows represent genes and the orientation of their transcription. Numbers indicate the corresponding MMP genes (e.g., 13 is *MMP13*). The drawing represents only the negative strand of the region. (**B** and **C**) Expression levels of MMP genes in human keratinocyte cells. HKerE6SFM cells were treated with UVB, TSA or UVB + TSA. The mRNAs were isolated 14 hours after the first treatment, and the expression levels of *MMP13*, *MMP12*, *MMP3*, *MMP1*, *MMP10* (**B**) and *MMP9* (**C**) were analysed by qPCR. The means and standard deviations of six independent experimental triplicates are indicated as fold-expression compared to the control. Star indicates statistical significance between the dataset (paired Anova *P < 0.05, **P < 0.01, ***P < 0.001, ****P < 0.0001). Legends for the X-axis are *control* (no treatment), *UVB* (only UVB no TSA), *TSA* (only TSA no UVB) and *UVB* + *TSA* (both UVB and TSA). (**D**) Pre-mRNA levels of *MMP3*. HKerE6SFM cells were treated with 80 mJ/cm^2^ UVB. The mRNAs were isolated 2, 8 and 24 hours after the first treatment, and pre-mRNA levels of MMP3 were analysed by qPCR. The means and standard deviations of five independent experimental samples are indicated as fold-expression compared to the control. Star indicates statistical significance between the dataset (paired Anova P values as indicated above).

Further analysis of the data revealed that metalloprotease-encoding genes was a group that was represented in significantly high numbers among the genes affected by the three treatment protocols. The expression of five matrix metalloprotease genes was activated in response to one or more stimuli. For example, the UVB and UVB + TSA treatments upregulated *MMP13*, *MMP12* and *MMP3*, while *MMP13* was also overexpressed slightly upon TSA treatment as compared to the control. *MMP1* and *MMP10* were upregulated only in UVB + TSA-treated cells. *MMP13* and *MMP12* showed superinduction in UVB + TSA-treated cells compared to those that were only UVB irradiated. Significantly, the genes encoding the five MMP proteins are located in one cluster on chromosome 11 together with four additional MMP genes (Fig. 2A). Interestingly, the five UVB-induced MMP genes are localized in close proximity forming a subgroup within the MMP locus on the 11q22.3 chromosome. According to the microarray data, the four additional MMP genes of the cluster (*MMP8*, *MMP27*, *MMP20* and *MMP7*) were either unaltered or expressed at low or undetectable levels under these conditions (data not shown).

### UVB alters the expression of MMP group genes in the 11q22.3 region

In order to verify the microarray data and further examine the role of histone acetylation during the UVB response of the chromosome 11 MMP gene cluster, we determined the mRNA levels of *MMP13*, *MMP12*, *MMP3*, *MMP1* and *MMP10* by qPCR following TSA treatment. The HKerE6SFM cells were first irradiated with UVB followed by TSA treatment as shown in row b in Fig. 1A. Although *MMP9* expression was not altered significantly in our microarray data, it was included in this study due to previous reports of its overexpression upon UVB irradiation of skin cells *in vivo*^17^ (Fig. 2 and Supplementary Fig. 2). The results of qPCR experiments verified the data obtained from our microarray experiment in that the MMP genes localized in the 11q22.3 region were upregulated in response to UVB irradiation. The mRNA levels of these genes determined by qPCR*—*in particular those of *MMP3* and *MMP12—*were higher than the expression levels indicated by the microarray data. This discrepancy could result from the different sensitivities of the two techniques (Fig. 2B, 2^nd^ bars of each gene). In accordance with the microarray data, *MMP9* mRNA levels were unaffected following UVB irradiation (Fig. 2C). Finally, we detected significant transcriptional induction of *MMP13*, *MMP12* and *MMP3*, but only modest activation of *MMP1* and *MMP10 *following the combined treatment of UVB and TSA (Fig. 2B, 4^th^bars of each gene).

To verify that the elevated mRNA levels were indeed consequences of elevated transcription triggered by UVB irradiation, we measured the pre-mRNA levels of *MMP3* at different time points after UVB irradiation of HKerE6SFM cells. The steady increase in *MMP3* pre-mRNA levels after irradiation indicated a UVB-dependent transcriptional upregulation (Fig. 2D) rather than the stabilization of *MMP3* mRNA.

**Figure 3:**
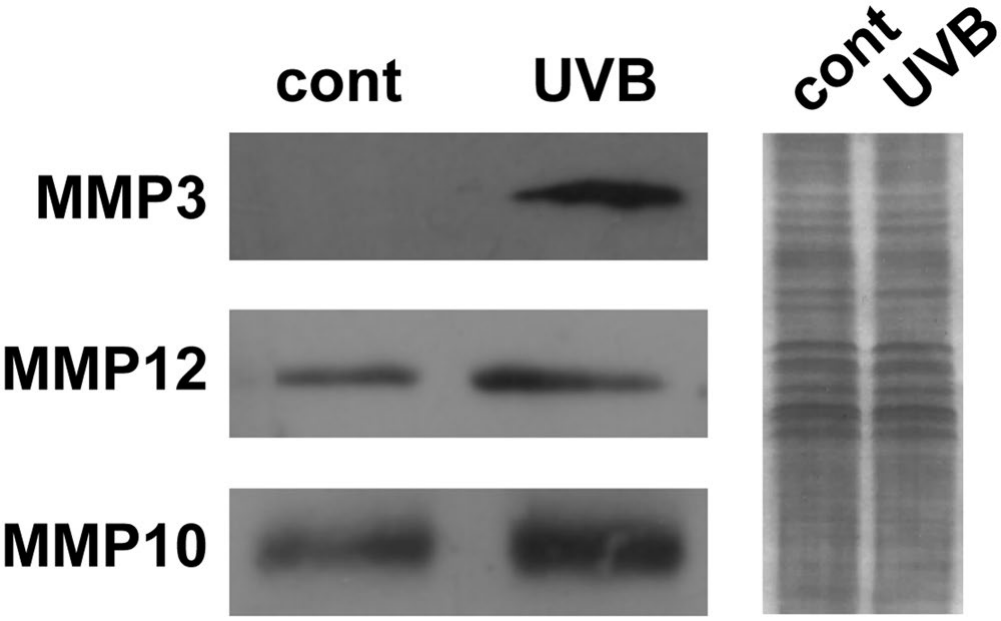
The protein levels of MMP3, MMP10 and MMP12. Protein levels were detected by Western blot analysis in control and UVB-treated HKerE6SFM cells. Loading controls are represented by Coomassie Brilliant Blue staining. The grouping of blots were cropped from different gels as shown in Supplementary Figure S2.

Next, we determined whether the elevated MMP mRNA levels correlated with higher MMP protein levels upon UVB treatment. Immunoblot assays were used to detect the levels of MMP3, MMP10 and MMP12 proteins in UVB-treated cells (Fig. 3 and Supplementary Fig. S3). In summary, these findings highlight the UVB-dependent upregulation of MMP gene expression at 11q22.3 and that the transcriptional activation of *MMP3*, *MMP10* and *MMP12* also correlates with higher MMP protein levels. The more robust gene activation following TSA treatment suggests that acetylation plays a role activating the MMP gene cluster, which may have a significant effect on the activation of *MMP13*, *MMP12* and *MMP3*.

To verify that histone acetyltransferases are required for UVB-triggered *MMP* gene activation, we performed histone acetyltransferase inhibitor (HATi) assays on HKerE6SFM cells. The simultaneous treatment by UVB and HATi correlated with lower MMP mRNA levels compared to cells treated with UVB alone (Fig. 2E and F). These data are consistent with our previous findings that histone acetylation maybe involved in the UVB-triggered transcriptional activation of the MMP cluster.

### RNAPII occupancy and the H3K9 acetylation pattern correlate with the activation of MMP genes at the 11q22.3 region

**Figure 4:**
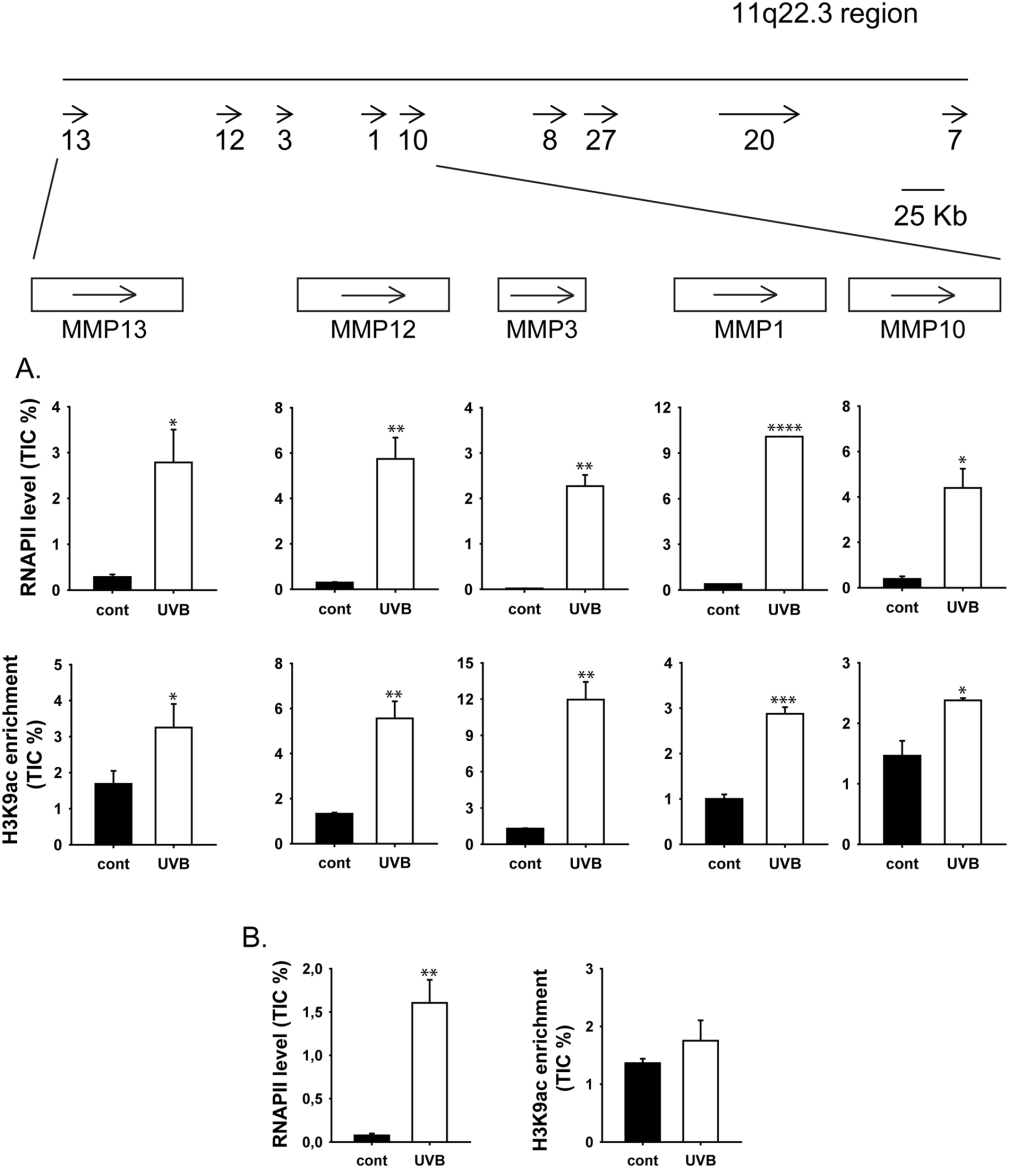
Recruitment of RNAPII and acetylated H3K9 on transcription initiation sites of MMP genes in human keratinocytes. HKerE6SFM cells were exposed to 80 mJ/cm^2^ of UVB irradiation or mock treated. Chromatin was prepared from each sample 24 hours after the treatment. Antibodies against RNAPII, H3 and acetylated H3K9 were used for the immunoprecipitation. Precipitated chromatin samples were analysed by qPCR. Enrichments of RNAPII, H3 and acetylated H3K9 were calculated as % of total inputs and normalized to the H3 content of control samples. The means, standard deviations and significance levels based on three independent experimental triplicates are indicated. Star indicates statistical significance between the dataset (paired Anova *P < 0.05, **P < 0.01, ***P < 0.001, ****P < 0.0001). (**A**) RNAPII and H3K9ac enrichments in transcription initiation sites of MMP genes encoded by the 11q22.3 genomic region. (**B**) RNAPII and H3K9ac enrichments of *MMP9* negative control gene.

Elevated levels of K9 acetylated H3 histone (H3K9ac) are frequently associated with active transcription and are enriched around the transcription start sites of actively transcribed genes^42^. To investigate the relationship between UVB irradiation and H3K9ac levels at the promoter regions of MMP genes, chromatin immunoprecipitation was used to measure H3K9ac levels at the promoters of members of the MMP gene cluster. In addition to H3K9ac levels, the density of RNAPII at the transcription initiation sites of *MMP13*, *MMP12*, *MMP3*, *MMP1* and *MMP10 *was determined. The *MMP9* gene was included in this analysis as a negative control due to the lack of effect of UVB irradiation on its mRNA levels. We observed a UVB-dependent increase in RNAPII binding around the transcription start site (TSS) of each MMP gene. The most pronounced change (approximately 110-fold increase) was observed at the *MMP3* TSS (Fig. 4A). On the contrary, RNAPII binding remained unchanged from that in the control intergenic region (data not shown) at the transcription initiation site of *MMP9* (Fig. 4B).

The elevations in acetylated H3K9 levels at transcription initiation sites were similar to RNAPII enrichment regions (Fig. 4A). The two least overexpressed genes *MMP13* and *MMP10* showed the lowest H3K9ac levels of 1.9- and 1.6-fold increases, respectively (Fig. 4A). In contrast, higher H3K9ac levels occurred at the initiation sites of the most highly induced *MMP12* and *MMP3* genes. As expected, H3K9ac levels remained unchanged at the transcription initiation site of *MMP9*. Thus, the density of acetylation marks is in good correlation with RNAPII enrichment and gene activation at this cluster of genes, suggesting that H3K9 acetylation plays an important role in the coordinated UVB response of MMP genes located in the 11q22.3 region.

## Discussion

Here, we report for the first time that UVB irradiation correlates with transcriptional activation at the11q22.3 genomic locus, which encodes a group of matrix metalloprotease proteins. The recently established immortalized human keratinocyte cell line, HKerE6SFM, was used. Several MMPs, such as *MMP1*, *MMP3*, *MMP9*, were previously reported as UV-responsive genes^16^. Our results demonstrated that the expression of not only these but nearly the entire MMP cluster became transcriptionally active 14 hours after UVB irradiation. On the other hand, *MMP9*, located on the 11^th^ chromosome represents a clear exception. Gene-specific histone acetylation has been associated with UVB-induced transcription of several genes in HaCaT cells^33^. Accordingly, we observed a further induction (i.e., superinduction) of *MMP13*, *MMP12* and *MMP3* upon combined treatment by UVB and TSA, indicating that histone acetylation plays a key role in the UVB-dependent activation of these genes. Chromatin immunoprecipitation experiments using an anti-H3K9ac antibody revealed a correlation between H3K9ac and MMP mRNA levels. Furthermore, RNA polymerase II occupancy showed a UVB-dependent increase at the same highly induced MMP promoter regions. These results suggest that there is a connection between the enrichment of H3K9ac and elevated RNAPII binding at the promoter regions of the MMP cluster. Together, these findings indicate that histone acetylation has an important role in the UVB response of MMP genes and that the density of the active gene histone mark, H3K9ac, determines the strength of the gene expression induction.

Cooper and colleagues recently reported the activation of the MAPK pathway during the cellular UV response, which leads to the formation of active NF-kappaB and AP-1 factors. Subsequently, AP-1 binds to its responsive elements at the matrix metalloprotease cluster^14^. Bioinformatic analysis performed by us and available public database (UCSC Genome Browser, https://genome.ucsc.edu) have revealed several AP-1 binding sites in the MMP cluster on chromosome 11. Additionally, Schmucker and colleagues have also demonstrated that the ILß-activation of MMP-13 is highly dependent on a distant AP-1 enhancer element in chondrocytes^43^. Our findings and these recently published data suggest a multistep activation process involving AP-1 binding and histone acetylation leading to the high transcriptional activation of the MMP cluster at the 11q22.3 region in response to UVB irradiation.

## Electronic supplementary material

Supplementary Information
