# Supplementary material for: Coordinated activation of a cluster of MMP genes in response to UVB radiation
Source: Sci Rep. 2018 Feb 8;8:2660. doi: 10.1038/s41598-018-20999-6 (PMC5805780; doi:10.1038/s41598-018-20999-6)
Supplement: Supplementary file 1 — Supplementary Information [file 41598_2018_20999_MOESM1_ESM.pdf]

Coordinated activation of a cluster of MMP genes in response to UVB radiation

Zsuzsanna Ujfaludi, Agota Tuzesi, Hajnalka Majoros, Balint Rothler, Tibor Pankotai, Imre M. Boros

Supplementary informations

Fig S1.

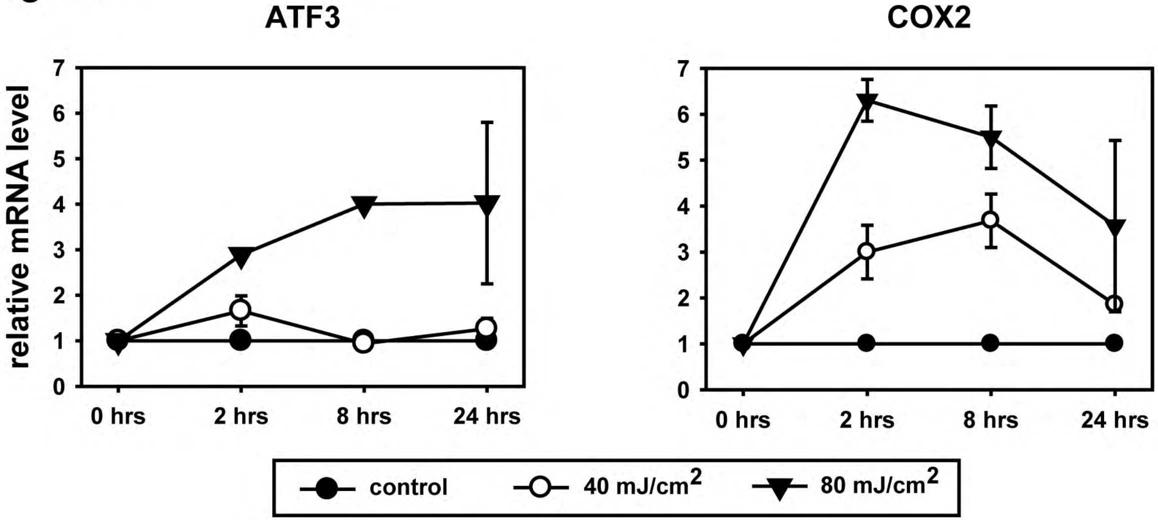

Supplementary figure 1.
Induction of ATF3 and COX2 in HKerE6SFM cells upon UVB irradiation.
HKerE6SFM cells were irradiated with 0, 40 or 80 mJ/cm2 of UVB. RNA was isolated 2, 8 and 24 hours after the exposure. Expression levels of ATF3 and COX2 were analyzed by QPCR. Means and standard deviations of three independent experimental triplicates are indicated as fold expression relative to control.

Fig S2.

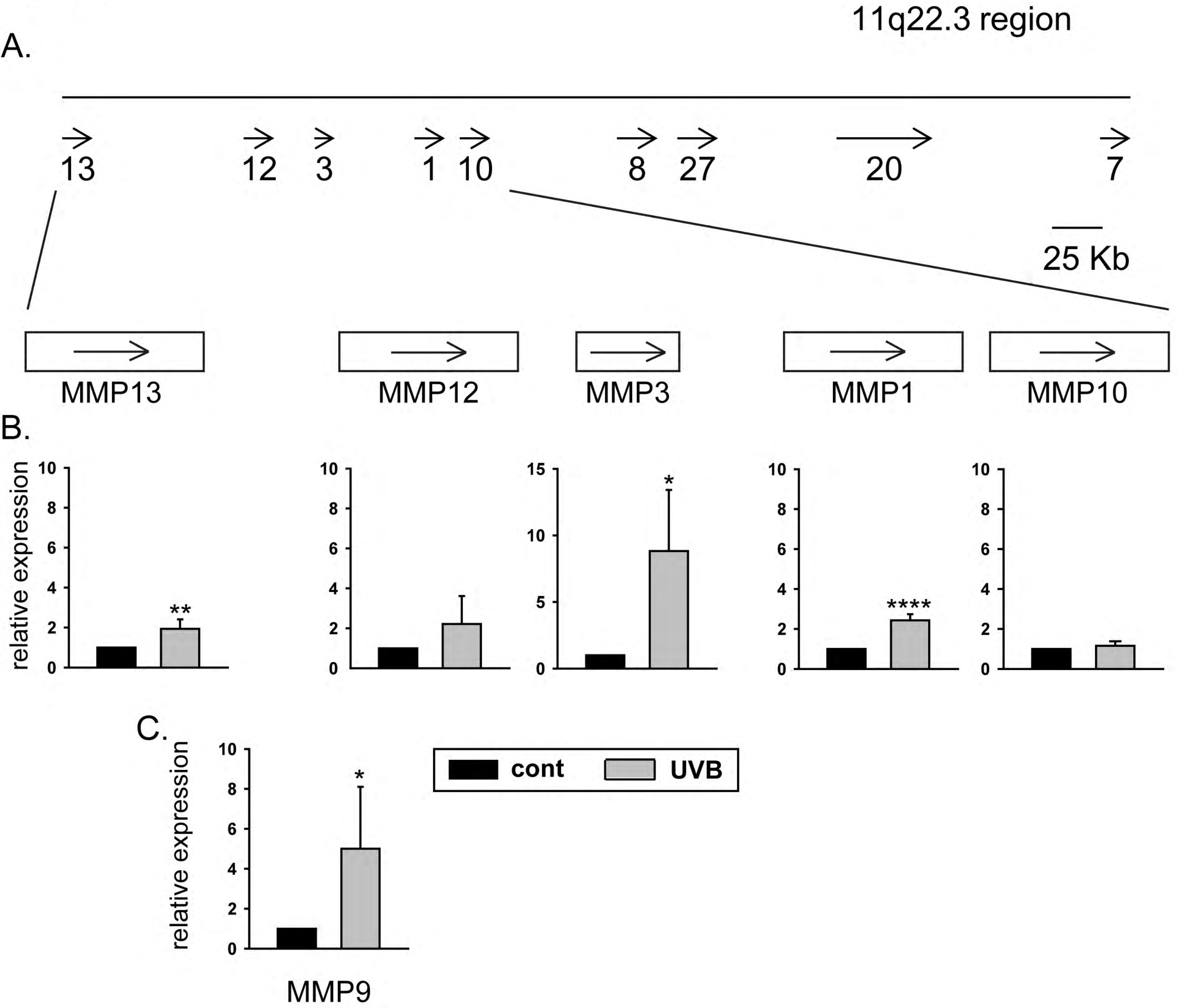

Supplementary figure 2.

Differential expression of MMP genes located in the 11q22.3 region and MMP9 in HaCaT cells.

A: Schematic drawing of the MMP gene cluster localized at chromosome 11q22.3 based on the NCBI GeneBank database. Arrows represent genes and the orientation of their transcription. Numbers indicate the corresponding MMP genes (e.g., 13 is MMP13). The drawing represents only the negative strand of the region. B and C: Expression levels of MMP genes in HaCaT cells. HaCaT cells were treated with UVB, TSA or UVB+TSA. The mRNAs were isolated 14 hours after the first treatment, and the expression levels of MMP13, MMP12, MMP3, MMP1, MMP10 (B) and MMP9 (C) were analysed by qPCR. The means and standard deviations of six independent experimental triplicates are indicated as fold-expression compared to the control. Legends for the X-axis are control (no treatment), UVB (only UVB no TSA), TSA (only TSA no UVB) and UVB+TSA (both UVB and TSA).

Fig S3.

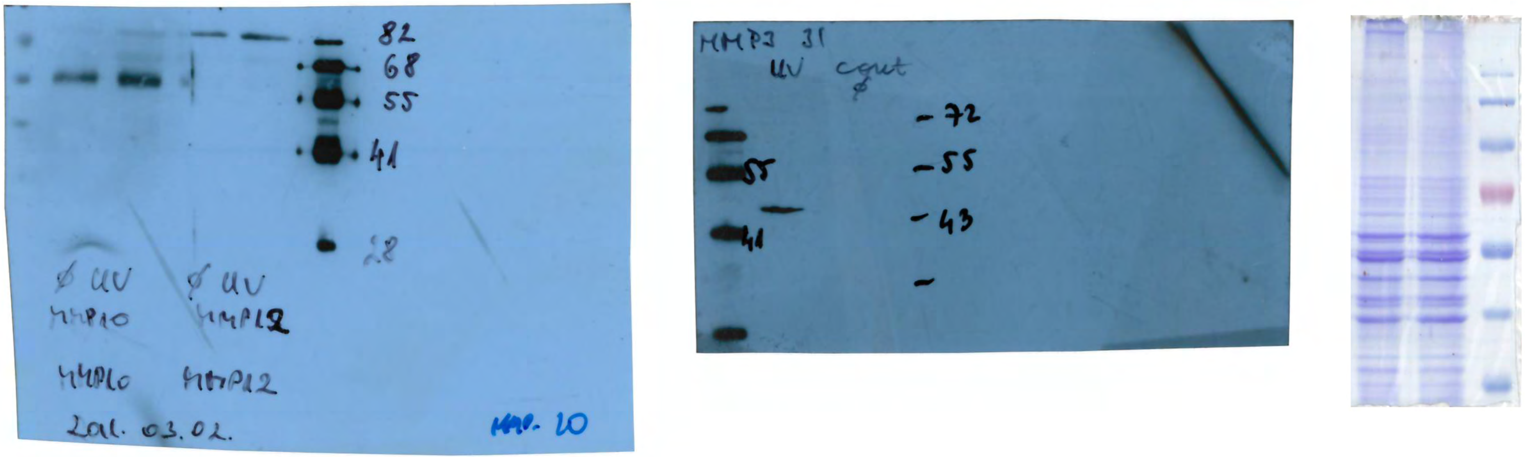
82
68
55
41
28
ø UV
MMP10
ø UV
MMP12
MMP10
MMP12
MMP3 31
UV
cont
ø
72
55
43
55
41
